# Supplementary material for: MetaRibo-Seq measures translation in microbiomes
Source: Nat Commun. 2020 Jun 29;11:3268. doi: 10.1038/s41467-020-17081-z (PMC7324362; doi:10.1038/s41467-020-17081-z)
Supplement: Supplementary file 10 — Supplementary Data 7 [file 41467_2020_17081_MOESM10_ESM.zip › File2/Confidence_High_Taxonomy/26347_out.krona.html]

Javascript must be enabled to view this page.

members
magnitude
magnitudeUnassigned
count
unassigned
taxon
rank

26347\_out

153

superkingdom
153
2

phylum
153
1239

class
153
186801

153
186802
order

186806
1
family

genus
1730
1

species

SRS051031\_contig\_number\_5312
1
39496

541000
152
2

SRS045826\_contig\_number\_contig-100\_25260.70840SRS077849\_contig\_number\_5659
family

149
11

SRS014235\_contig\_number\_41809SRS017307\_contig\_number\_8880SRS024132\_contig\_number\_42003SRS064232\_contig\_number\_contig-100\_315.316SRS077730\_contig\_number\_contig-100\_1156.75790SRS078419\_contig\_number\_contig-100\_1298.82629SRS1041142\_contig\_number\_4573SRS143372\_contig\_number\_contig-100\_7657.46623SRS144506\_contig\_number\_2547SRS147425\_contig\_number\_contig-100\_10765.51063SRS893170\_contig\_number\_3785
genus
1263

2293203
7

SRS014287\_contig\_number\_16165SRS014855\_contig\_number\_6109SRS015854\_contig\_number\_15934SRS051882\_contig\_number\_13337SRS077502\_contig\_number\_17725SRS104084\_contig\_number\_6802SRS144135\_contig\_number\_7594
species


SRS098881\_contig\_number\_24959SRS144753\_contig\_number\_591SRS146813\_contig\_number\_contig-100\_614.163083
species
1897020
3

species

SRS097889\_contig\_number\_39996SRS1055056\_contig\_number\_6277SRS143780\_contig\_number\_32453
3
2293209

10
2293148
species

SRS011239\_contig\_number\_13270SRS016954\_contig\_number\_591SRS018656\_contig\_number\_1995SRS018936\_contig\_number\_3431SRS049773\_contig\_number\_944SRS075021\_contig\_number\_2062SRS077194\_contig\_number\_1131SRS1041036\_contig\_number\_1507SRS143523\_contig\_number\_15307SRS893279\_contig\_number\_16586

1262950
7

SRS017821\_contig\_number\_30604SRS018817\_contig\_number\_contig-100\_559.137368SRS050026\_contig\_number\_11741SRS098655\_contig\_number\_5403SRS1041136\_contig\_number\_3511SRS143417\_contig\_number\_14058SRS147557\_contig\_number\_3081
species

20
2293179
species

SRS014948\_contig\_number\_23782SRS018427\_contig\_number\_15378SRS019030\_contig\_number\_4147SRS019381\_contig\_number\_7587SRS019445\_contig\_number\_16492SRS020328\_contig\_number\_9342SRS024492\_contig\_number\_8925SRS042966\_contig\_number\_17234SRS043411\_contig\_number\_17074SRS049446\_contig\_number\_10693SRS053214\_contig\_number\_13722SRS054956\_contig\_number\_2696SRS062701\_contig\_number\_899SRS075878\_contig\_number\_5811SRS077335\_contig\_number\_3553SRS098644\_contig\_number\_contig-100\_84.279521SRS1041129\_contig\_number\_2514SRS105153\_contig\_number\_31557SRS140492\_contig\_number\_1919SRS147919\_contig\_number\_6653


SRS013476\_contig\_number\_28915SRS014923\_contig\_number\_42139SRS015578\_contig\_number\_34736SRS050925\_contig\_number\_contig-100\_187.197884SRS064757\_contig\_number\_811SRS077294\_contig\_number\_13158SRS147271\_contig\_number\_31132SRS893369\_contig\_number\_5021
species
2293206
8

species

SRS011302\_contig\_number\_26322SRS012273\_contig\_number\_5147SRS013158\_contig\_number\_2304SRS013687\_contig\_number\_1073SRS013965\_contig\_number\_27444SRS015190\_contig\_number\_contig-100\_461.98375SRS015663\_contig\_number\_2019SRS015960\_contig\_number\_15029SRS016018\_contig\_number\_2100SRS016203\_contig\_number\_contig-100\_90.78795SRS017701\_contig\_number\_18036SRS017916\_contig\_number\_12745SRS018351\_contig\_number\_1053SRS018623\_contig\_number\_1947SRS018836\_contig\_number\_11508SRS019068\_contig\_number\_115964SRS019601\_contig\_number\_contig-100\_592.162078SRS019808\_contig\_number\_contig-100\_1316.107755SRS019968\_contig\_number\_9276SRS020233\_contig\_number\_24871SRS020394\_contig\_number\_13104SRS022071\_contig\_number\_1701SRS023914\_contig\_number\_1743SRS023971\_contig\_number\_3975SRS024331\_contig\_number\_37868SRS042628\_contig\_number\_16273SRS045739\_contig\_number\_8369SRS046369\_contig\_number\_11886SRS048981\_contig\_number\_4627SRS050998\_contig\_number\_contig-100\_1284.81063SRS054905\_contig\_number\_16384SRS056259\_contig\_number\_contig-100\_367.171090SRS056273\_contig\_number\_9361SRS063190\_contig\_number\_12693SRS063985\_contig\_number\_29244SRS064276\_contig\_number\_19772SRS065504\_contig\_number\_16920SRS075821\_contig\_number\_contig-100\_111.125406SRS075984\_contig\_number\_26405SRS077641\_contig\_number\_14695SRS078242\_contig\_number\_31585SRS097958\_contig\_number\_4997SRS1041033\_contig\_number\_5509SRS1041039\_contig\_number\_689SRS1041137\_contig\_number\_23277SRS104400\_contig\_number\_7574SRS104912\_contig\_number\_19015SRS1055043\_contig\_number\_441SRS1055099\_contig\_number\_13312SRS142542\_contig\_number\_14390SRS142923\_contig\_number\_13147SRS143895\_contig\_number\_contig-100\_510.253512SRS144183\_contig\_number\_1642SRS147346\_contig\_number\_10137SRS147977\_contig\_number\_2649SRS148196\_contig\_number\_17578SRS148424\_contig\_number\_21644SRS148817\_contig\_number\_574SRS892882\_contig\_number\_14028SRS893373\_contig\_number\_20681SRS893383\_contig\_number\_29441
61
40518

1
2293195
species

SRS016132\_contig\_number\_4628

2
165186
species

SRS011084\_contig\_number\_7543SRS021484\_contig\_number\_31914

41978
16

SRS012969\_contig\_number\_contig-100\_50.241597SRS015431\_contig\_number\_70001SRS016495\_contig\_number\_16114SRS017859\_contig\_number\_6255SRS018541\_contig\_number\_12669SRS023346\_contig\_number\_6529SRS050422\_contig\_number\_contig-100\_28.197011SRS053335\_contig\_number\_2353SRS1041139\_contig\_number\_5685SRS146812\_contig\_number\_13926SRS148159\_contig\_number\_3524SRS148319\_contig\_number\_8732SRS148721\_contig\_number\_56093SRS148874\_contig\_number\_27SRS149075\_contig\_number\_10203SRS971275\_contig\_number\_contig-100\_171.262312
species

genus
1
292632

1
1262970
species

SRS023526\_contig\_number\_1303
